# Examining Employment Conditions During the COVID-19 Pandemic in Pasifika Communities

**DOI:** 10.1089/heq.2022.0027

**Published:** 2022-08-10

**Authors:** Santino G. Camacho, Kilohana Haitsuka, Kenneth Yi, Joseph Seia, David Huh, Michael S. Spencer, David Takeuchi

**Affiliations:** ^1^ University of Washington School of Social Work, Seattle, Washington, USA.; ^2^ Indigenous Wellness Research Institute, Ola Pasifika, Seattle, Washington, USA.; ^3^ Pacific Islander Community Association of Washington, Federal Way, Washington, USA.

**Keywords:** COVID-19, Pacific Islander, NHPI, settler colonialism, employment, socioeconomic health

## Abstract

**Introduction:**

Pasifika (Native Hawaiian and Pacific Islander) people living in the United States experience health, economic, and social inequities, and a disproportionate burden of COVID-19 cases and deaths. This study examines employment among Pasifika living in the 10 US states with the largest Pasifika populations during the COVID-19 pandemic.

**Methods:**

We use the Current Population Survey to examine racial differences in employment status, paid work from home (PWFH), and industry telework friendliness. We use data from the Washington Office of Fiscal Management and the Washington State (WA) Employment Security Department to examine county-level unemployment claims.

**Results:**

Nationally, Pasifika did not self-report unemployment significantly more than Black, Latino, Asian, and American Indian/Alaska Native respondents, but in WA counties with high Pasifika concentrations, unemployment insurance claim rates were higher compared with all other racial groups, particularly Whites and Asians. Surprisingly, Pasifika had more PWFH opportunities, but worked in less telework-friendly industries nationally.

**Discussion:**

This study demonstrates the complexity of employment among Pasifika during the COVID-19 pandemic. The findings correspond with national reports of racialized communities impacted by unemployment, including Pasifika. Marginally significant differences in unemployment nationally may be due to Pasifika working largely in essential industries requiring workplace attendance.

**Health Equity Implications:**

Although overlooked or overshadowed by size, our findings highlight the need for continued advocacy to support data disaggregation and Pasifika data sovereignty. This can be achieved through collaborations between researchers as well as local and community organizations to address data needs of Pasifika communities.

## Introduction

In the United States (US), structural racism and settler colonialism drive the health disparities experienced by Pasifika (Native Hawaiian and Pacific Islander [NH/PI]) communities and frame the concern for and a focus on Pasifika populations in this article. Militourism—the exploitation of Indigenous land, people, and resources through the synergistic relationship between US military expansion and the tourism industry—and other settler colonial systems^1–7^ have historically produced negative outcomes for the peoples and lands of the Pacific, including inequities in health outcomes, displacement of Pasifika from their homelands, and persistent poverty and homelessness.^2,4,6,8–15^ However, while there is a shared history among Pasifika, the racialization of Pasifika in the US is complex in that the definition of indigeneity varies between Pasifika communities depending on their political relationship with the US government.

Diversity in citizenship, migration status, language, and cultures among Pasifika poses additional challenges to research with Pasifika populations. In this article, we use Pasifika and Native Hawaiian/Pacific Islander interchangeably. Pasifika was an agreed-upon term that the research team and community partners felt was culturally appropriate, honored Pasifika data sovereignty, and was inclusive of Native Hawaiians and Pacific Islanders from Micronesia, Melanesia, and Polynesia. NH/PI is used when data are defined as this category.

Socioeconomic status (SES) is a well-established social determinant of health and is inextricably rooted in structural racism and settler colonialism in the US.^16–26^ Employment and occupational status are common and well-documented indicators for SES as a social determinant of health.^27,28^ As an indicator of socioeconomic wellness, employment can be a proxy for understanding financial security as well as access to health insurance and in turn health care.^28,29^ The operationalization and utilization of SES depend on the political and social contexts in which they are used.^29–32^ Within the context of the COVID-19 pandemic, the impact of lost wages due to illness/death, one's inability to work from home, immunovulnerability due to chronic health problems, and work in essential industries require us to look beyond income or employment alone.

In 2019, the US Census Bureau reported that 14.8% of NH/PI were living at the poverty level compared with 9.0% of non-Latino Whites, and the unemployment rate for NH/PI was 5.9% compared with 3.7% for non-Latino Whites.^33^ Thus, inequities related to SES in addition to pandemic-related factors make Pasifika potentially more vulnerable to negative health outcomes. Data for Washington state (WA) are relevant because WA has the third largest population of NH/PI living in the US, outside of Hawaii and California.^33^ According to the University of California, Los Angeles (UCLA) Center for Health Policy Research, as of March 10, 2022, NH/PI living in WA state have the highest per capita case (29,456 cases per 100,000) and death (297.2 cases per 100,000) rates of COVID-19 among all racial/ethnic groups.^4^ Furthermore, in King county from 2015 to 2019, 23.2% of NH/PI lived at or below the 200% poverty line, which is ∼14% higher than that of Asians and 19% than that of Whites.^34^

Since the start of the pandemic, Pasifika living across the US have experienced COVID-19 cases, hospitalizations, and deaths at disproportionate rates compared with Whites and other racial/ethnic minority communities.^33,35–37^ Across all ages, mortality from COVID-19 for NH/PI (12.7%) was significantly higher compared with Whites (6.5%).^38^ Pasifika COVID-19 mortality remains high in states with large populations of Pasifika. For example, as of March 10, 2022, the UCLA Center for Health Policy Research noted that NH/PI have the highest rates of COVID-19 death rates in Hawaii (128.2 cases per 100,000), California (365.2 cases per 100,00), and Utah (235.1 cases per 100,000).^37^ These disparities are reflective of historical and existing systemic inequities that were exacerbated by the COVID-19 pandemic.^39–42^

This study seeks to identify potential socioeconomic inequities experienced by Pasifika in 10 US states. Only a few studies have focused on the relationship between Pasifika and COVID-19,^38,39,42–44^ and this is the first to examine the socioeconomic impact of COVID-19 on Pasifika communities.

## Methods

Our work was guided by participatory research principles and our community partner was the Pacific Islander Community Association of Washington (PICA WA). Through this partnership, we support PICA WA's efforts to provide timely and culturally relevant health, economic, and social services within the state of WA and national efforts to promote NH/PI health equity research through organizations, such as the National Association of Pasifika Organizations (NAOPO).

**FIG. 1.**
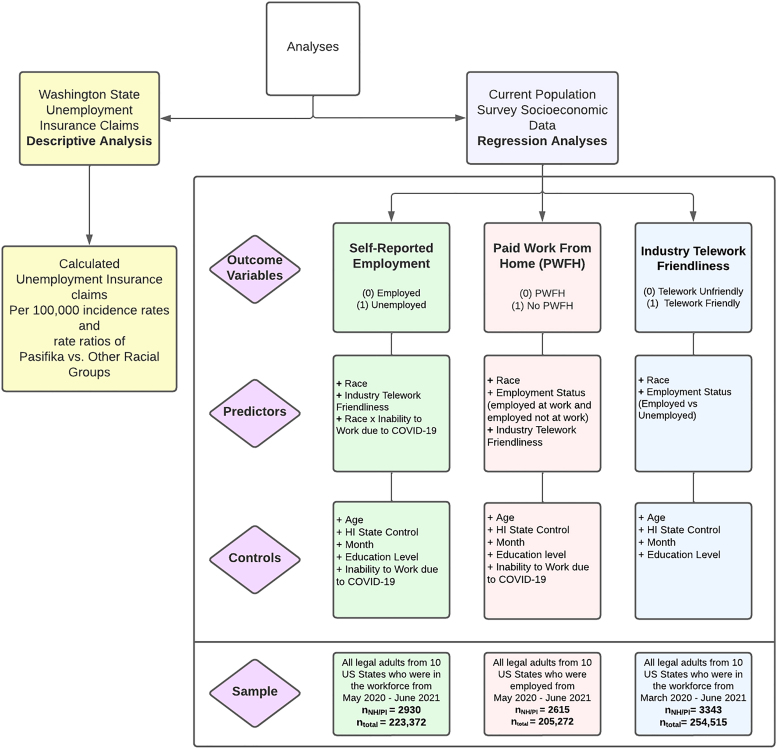
Analysis guide for all statistical analyses.

WA socioeconomic and demographic data were collected from the WA Office of Fiscal Management (OFM) and the WA Employment Security Department (ESD). National socioeconomic and demographic data were accessed from the Current Population Survey (CPS)—a demographics and labor survey conducted by the Bureau of Labor Statistics and the US Census Bureau. CPS data were accessed through the Integrated Public Use Microdata Series’ (IPUMS) CPS database managed by the Minnesota Population Center located at the University of Minnesota.^45^ IPUMS CPS contains integrated microdata from the CPS that are publicly available. No IRB approval was required because data were gathered from public data sources. We conducted two sets of analyses, one descriptive for the WA state data, and one set of three logistic regressions for our national data (Fig. 1).

### WA unemployment data

To examine the impact of COVID-19 on Pasifika employment in WA, we calculated per capita incidence rates to examine the differences in unemployment insurance claims made between Pasifika and other racial groups for Snohomish, Spokane, Clark, Pierce, and King County and for the state of WA. Incidence rates were calculated using the total number of unemployment insurance claims data by race between March 2020 and January 2022 collected by the ESD.^46^ We divided the initial claims by the 2020 race census data available through the OFM.^47^ We then calculated per 100,000 incidence rates for select counties by dividing initial claims by census for NH/PI, non-Latino White, Black, Asian, Latino, American Indian, and Alaska Native (AI/AN), and Multiracial groups for select counties. Counties were selected for analyses based on areas highly serviced by PICA WA and population density of PI's living within each respective county.

### National employment and COVID-19-related SES

Using the CPS, we conducted a series of three separate logistic regressions to examine the employment conditions of COVID-19 for racial groups compared with Pasifika on the following outcomes: (1) self-reported unemployment (n_NH/PI_=2930, n_total_=223,372), (2) paid work from home (PWFH) (n_NH/PI_=2615, n_total_=205,272), and (3) whether they worked in a telework-friendly industry (n_NH/PI_=3343, n_total_=254,515) (Fig. 1). A handful of samples of CPS data were taken from the IPUMS CPS data repository predicated on the restrictions of the outcome variable parameters (see details in Fig. 1). All samples included legal adults from the 10 states with the highest population counts of NH/PI among US states (Hawaii, Utah, Washington, Florida, Texas, California, Nevada, Oregon, New York, and Arizona).^33^ This was done to adjust for a small sample proportion of Pasifika living in the US compared with overall sample sizes.

All models were controlled for age (in years), time (months data were collected), education level (level of education from no education to graduate or professional degree), and disproportionate sample size based on geographic location (Hawaii vs. not in Hawaii). The time frames from which data were selected for each model reflect the implementation of public health safety regulations on social distancing, business closures at the beginning of the COVID-19 pandemic, and data restrictions of COVID-19-related questions that were included in the CPS. The right side of Figure 1 defines each logistic regression model produced for this study and defines specific sampling parameters for each model.

#### Race/Ethnicity

Race/Ethnicity was developed utilizing microdata on racial and ethnic demographics that include up to four selections of race and Latino origin. To account for small sample size issues, we constructed a NH/PI category to include people who self-identified as NH/PI in the CPS alone or with any combination other races or ethnicities. All other racial groups were categorized into non-Latino monoracial groups with an additional non-Latino multiracial category created for individual comparisons in our regression. All individuals who identified as non-Latino Whites and all individuals who identified as Latino were categorized into distinct groups. Associations with race and the outcome variables were tested in separate logistic regression analyses. Following antiracist and decolonizing data science practices in studying health and social disparities, NH/PI served as the control group for all regression analyses.

#### Self-reported employment

Self-reported employment was our first outcome of interest. Individuals noted whether they were employed (at work, had a job but were not at work last week), unemployed (unemployed workers who have had a job, unemployed workers who have not yet had a job), and Not in the Labor Force (NILF—unable to work, not in labor force for another reason, or retired) at the time of survey. Different variations of these categorizations of self-reported employment were utilized based on practicality and sampling restrictions of employment categorizations in relation to outcome variables. We analyzed self-reported employment status' association with industry telework friendliness. In these model individuals who were NILF were not included. We also tested the relationship between ability to work due to COVID-19 (able to work vs. unable to work due to COVID-19 business closure or loss in the last 4 weeks) and self-reported employment status and its interaction with race.

We included ability to work due to COVID-19 in our model as a sensitivity analysis to address its impact on the relationship between race and employment to account for COVID-19's direct impact on employment stability. Specifically, we sought to account for how the pandemic altered people's ability to work and how this impacted overall employment during the pandemic. Data from May 2020 until June 2021 were available and included in this model.

#### Paid work from home

Paid work from home (PWFH) was our second outcome of interest. To assess whether someone was employed in a job that offered pay while working from home, we utilized a variable from the CPS that indicated whether someone worked from home due to COVID-19 in the last 4 weeks. This variable was only asked in the CPS to individuals who indicated that they were employed for pay. Due to this sampling frame, our sample only included employed participants. Data from May 2020 until June 2021 were available and included in this model. Data collection on COVID-19-related measurements within the CPS began in May 2020.

#### Industry telework friendliness

Industry telework friendliness was a categorical variable we created that divided US industries by telework friendliness—ability for jobs within an industry to accommodate telework—based on findings from Dalton^48^ and Dingel and Neiman,^49^ as well as employment amidst COVID-19. Individuals who worked in leisure and hospitality, retail trade, construction, transportation and warehousing, and manufacturing were categorized as working in industries that were telework unfriendly and all other industries were categorized as being telework friendly. More specifically, industries that were found nationally to have lower telework friendliness in Dalton^48^ and Dingel and Neiman^49^ were classified as telework unfriendly and those that were found to have higher telework friendliness were classified as telework friendly.

In this model, individuals who were NILF were not included in the sample. Additionally, we tested industry telework friendliness's association with self-reported employment. Data from March 2020 until June 2021 were available and included in this model.

All data cleaning, visualization, descriptive summaries, and regression modeling were conducted in R statistical computing software and Microsoft excel. All analyses, cleaning, summarization, and visualization were completed by a team led by Pasifika scholars. Asian scholar allies also supported the interpretation of the study findings.

## Results

### WA state unemployment insurance claims

WA data on NH/PI unemployment and census data allowed us to calculate incidence rates for unemployment insurance claims filed since March 2020. NH/PI have had the highest unemployment insurance claims incidence rates among all other racial groups since March 2020. Most interestingly, NH/PI had a 1.85 times higher incidence and 2.23 times higher incidence of unemployment insurance claims compared with Whites and Asians, respectively. NH/PI also had higher initial unemployment insurance claim rates compared with Whites and Asians in select counties where there were large populations of NH/PI (Table 1). NH/PI had approximately double the amount of unemployment insurance claims compared with Whites and Asians.

**Table 1.** Washington State Initial Unemployment Insurance Claims Incidence Rates Pasifika Versus Whites and Pasifika Versus Asians from March 8, 2020 to January 1, 2022

County	Pasifika initial claims per 100,000	Whites initial claims per 100,000	Rate ratio Pasifika:Whites	Asian initial claims per 100,000	Rate ratio Pasifika:Asians
Spokane	32,548.06	17,213.40	1.892	14,772.81	2.205
Snohomish	40,046.59	20,599.04	1.944	17,832.50	2.246
Clark	22,784.19	14,002.37	1.627	11,295.68	2.017
Pierce	32,059.65	19,616.75	1.634	18,037.05	1.777
King	32,218.13	17,976.66	1.792	14,080.70	2.288
Statewide	34,471.88	18,625.18	1.851	15,458.70	2.230

### National employment statistics

**FIG. 2.**
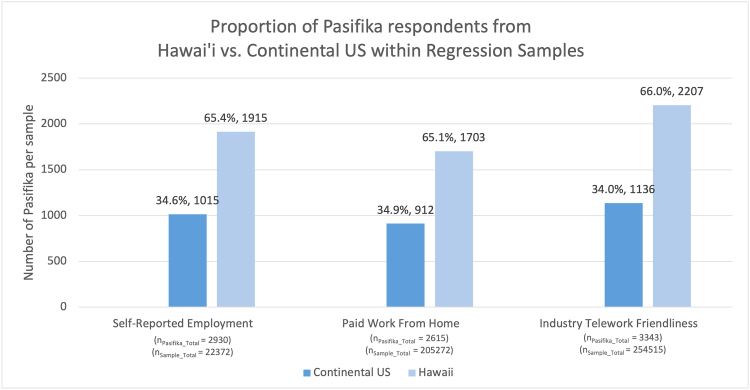
Pasifika sample size proportions by location for each logistic regression model samples.

Figure 2 displays the proportion of NH/PI living in Hawaii and in the Continental US across our three logistic regressions and presents the respective sample sizes for each regression. This was included to demonstrate sample bias by geolocation and highlight our decision to include a control variable for respondent's location (Hawaii vs. Continental U.S.). Our first logistic regression model analyzed the associations between race and self-reported employment status.

#### Employment

Table 2 summarizes the associations in odds ratios from our logistic regression between race and self-reported employment status.

**Table 2.** Logistic Regression of Self-Reported Employment Status

Variable	IV: (0)—Employed (1)—Unemployed		
	OR	CI 95%	***p***
Intercept	0.19	0.16–0.24	<0.001^***^
Race: White	0.84	0.71–1.01	0.065
Race: Asian	0.95	0.79–1.15	0.617
Race: Black	1.46	1.22–1.78	<0.001^***^
Race: AI/AN	1.67	1.26–2.21	<0.001^***^
Race: Hispanic any race	1.03	0.86–1.24	0.776
Race: multiracial	1.09	0.85–1.39	0.500
Industry: telework friendly	0.80	0.77–0.83	<0.001^***^
Age	0.99	0.99–0.99	<0.001^***^
Location: Hawaii	1.07	0.99–1.16	0.106
Month	0.99	0.99–1.00	0.001^**^
Education level	0.87	0.86–0.88	<0.001^***^
Unable to work due to COVID-19	10.58	8.18–13.71	<0.001^***^
Race: White×unable to work due to COVID-19	0.94	0.72–1.22	0.641
Race: Asian×unable to work due to COVID-19	1.28	0.97–1.68	0.083
Race: Black×unable to work due to COVID-19	0.80	0.61–1.06	0.128
Race: AI/AN×unable to work due to COVID-19	0.61	0.38–0.97	0.038^*^
Race: Hispanic×unable to work due to COVID-19	0.83	0.64–1.08	0.166
Race: multiracial×unable to work due to COVID-19	0.78	0.53–1.14	0.195
Observations	223,372		
R2 Tjur	0.129		

^***^ *p*<0.001, ^**^p<0.01, ^*^p<0.05.

AI/AN, American Indian and Alaska Native; CI, confidence interval; OR, odds ratio.

When comparing employment status between NH/PI and other racial ethnic groups, we found that AI/AN respondents had a significantly higher odds of reporting that they were unemployed compared with NH/PI respondents who were able to work during COVID-19. AI/AN had a 67% higher odds (odds ratio [OR]=1.67, *p*<0.001, 95% confidence interval [CI] [1.26–2.21]) of self-reporting unemployment compared with NH/PI respondents. Black respondents had a 46% higher odds (OR=1.46, *p*<0.001, 95% CI [1.22–1.78]) of self-reporting unemployment compared with NH/PI respondents. Whites had a marginally significant, 16% lower odds (OR=0.84, *p*=0.062, 95% CI [0.79–1.15]) of self-reporting unemployment compared with NH/PI respondents. No significant difference was found among NH/PI, Asians, and Latinos. The association between AI/AN and self-reported unemployment was smaller among those that were unable to work (OR=0.61, *p*<0.05, 95% CI [0.38–0.97]).

#### Paid work from home

Table 3 summarizes the associations in odds ratios from our logistic regression between race and whether CPS respondents reported having PWFH.

**Table 3.** Logistic Regression of Paid Work from Home

Variable	IV: (0) PWFH (1) No PWFH		
	ORs	CI 95%	***p***
Intercept	13.47	11.74–15.47	<0.001^***^
Race: White	1.15	1.03–1.29	0.014^*^
Race: Asian	0.88	0.78–0.98	0.024^*^
Race: Black	1.48	1.31–1.67	<0.001^***^
Race: AI/AN	1.04	0.86–1.26	0.668
Race: Latino	1.61	1.44–1.81	<0.001^***^
Race: multiracial	0.91	0.79–1.04	0.174
Employed: employed not at work	2.43	2.28–2.60	<0.001^***^
Industry: telework friendly	0.54	0.53–0.55	<0.001^***^
Age	1.01	1.00–1.01	<0.001^***^
Location: Hawaii	1.58	1.48–1.68	<0.001^***^
Education level	0.57	0.57–0.58	<0.001^***^
Month	1.08	1.07–1.08	<0.001^***^
UNAW: unable to work due to COVID	0.70	0.67–0.73	<0.001^***^
Observations	205,272		
R2 Tjur	0.168		

^***^ *p*<0.001, ^**^p<0.01, ^*^p<0.05.

PWFH, paid work from home; UNAW, ability to work due to COVID.

Compared with NH/PI respondents White, Black, and Latino respondents had higher odds of reporting no PWFH. Surprisingly, White respondents had a 15% higher odds (OR=1.15, *p*<0.05, 95% CI [1.03–1.29]) of having no PWFH compared with NH/PI respondents. Black respondents had a 48% higher odds (OR=1.48, *p*<0.001, 95% CI [1.31–1.67]) of having no PWFH than NH/PI respondents. Latino respondents had a 61% higher odds (OR=1.61, *p*<0.001, 95% CI [1.44–1.81]) of having no PWFH compared with NH/PI. In contrast, Asians had a 12% lower odds (OR=0.88, *p*<0.05, 95% CI [0.78–0.98]) of having no PWFH compared with NH/PI.

#### Industry telework friendliness

Table 4 summarizes the logistic regression examining the association in odds ratios between race and employment in a telework-friendly industry.

**Table 4.** Logistic Regression of Telework Friendliness

Variable	IV: (0) telework-unfriendly industry, (1) telework-friendly industry		
	ORs	CI 95%	***p***
Intercept	0.54	0.49–0.59	<0.001^***^
Race: White	1.08	1.00–1.17	0.041^*^
Race: Asian	0.87	0.80–0.94	<0.001^***^
Race: Black	1.11	1.03–1.21	0.010^**^
Race: AI/AN	1.46	1.28–1.67	<0.001^*^
Race: Latino	1.00	0.92–1.08	0.926
Race: multiracial	1.06	0.96–1.17	0.279
Employment: unemployed	0.67	0.65–0.68	<0.001^***^
Age	1.00	1.00–1.00	<0.001^***^
Location: Hawaii	1.12	1.07–1.17	<0.001^***^
Month	1.00	1.00–1.00	0.004^**^
Education level	1.29	1.28–1.29	<0.001^***^
Observations	254,515		
R2 Tjur	0.045		

^***^ *p*<0.001, ^**^p<0.01, ^*^p<0.05.

When comparing NH/PI to other racial groups, we found that White, Black, and AI/AN respondents had higher odds than NH/PI of working in industries that were classified as telework friendly when controlling for employment status, age, location, month of response, and education level. White respondents had an 8% higher odds (OR=1.08, *p*<0.05, 95% CI [1.00–1.17]) of working in a telework-friendly industry compared with NH/PI. Black respondents had an 11% higher odds (OR=1.11, *p*<0.05, 95% CI [1.03–1.21]) of working in a telework-friendly industry compared with NH/PI. AI/AN respondents had a 46% higher odds (OR=1.46, *p*<0.001, 95%CI [1.28–1.67]) of working in a telework-friendly industry compared with NH/PI respondents. Notably, Asian respondents had a 13% lower odds (OR=0.87, *p*<0.001, CI [0.80–0.94]) of being employed in a telework-friendly industry compared with NH/PI respondents.

## Discussion

This study aimed to understand racial differences in employment-related outcomes during the COVID-19 pandemic with specific attention to Pasifika due to their disproportionate rates of COVID-19 infection, hospitalizations, deaths, and histories of settler colonialism that make them more vulnerable to negative outcomes related to the pandemic. Although several studies have focused on the impacts of COVID-19 on employment for other Communities of Color, none of them have focused on the employment outcomes of Pasifika nor have centered Pasifika as the comparison group.^50–52^

Our findings indicate that Pasifika were at lower odds for unemployment nationally compared with Black and AI/AN respondents in the labor force during COVID-19. However, Pasifika had a marginally higher odds of being unemployed compared with Whites nationally. Pasifika in WA had a higher incidence rate of unemployment insurance claims compared with other racial groups statewide and in counties where Pasifika are most concentrated. Larger margins of difference in WA unemployment insurance claims were noted for Pasifika compared with Asians and Whites.

Surprisingly, on a national level among employed survey respondents, Pasifika had more opportunities for PWFH compared with other racial groups, except for Asians. However, Pasifika did have lower odds of working in telework-friendly industries compared with White, Black, and AI/AN respondents. Interestingly, Asians were less likely than Pasifika to work in telework-friendly industries, which might also counter some stereotypes of Asian Americans dominating tech and other telework-friendly industries.

These findings have several implications. First, it is important to return to a structural racism and settler colonial lens. Educational attainment is generally lower among Pasifika and Pasifika employment has largely been dominated by low-paying jobs and work in industries that require minimal education (e.g., grocery, farming, shipping, construction, service industries, military, tourism, etc.).^33,39,42^ Pasifika are also more likely to live in multigenerational households, which can factor into increased COVID-19 transmission and mortality.^39,42^ During the pandemic, many of these jobs were deemed essential, meaning less risk for unemployment. In fact, early in the pandemic, Pasifika scholars noted that 24% of NH/PI worked in essential jobs.^39,42^

While national data demonstrate that Pasifika were able to work for pay from home, these data were only asked of those employed. The observed relationships may also be impacted by the quality of the data. Unemployment insurance claims, which state and federal departments utilize to measure employment in the US, may be stronger indicators of employment status than self-reported unemployment used in the CPS.

A major limitation of this article and its data is its inability to examine subgroups within the Pasifika population. Disaggregation of Pasifika from the Asian/Pacific Islander (API) category highlights important differences between Pasifika and Asian Americans and is an important first step in this study.^53^ In fact, our results highlighted the differences between NH/PI and Asian Americans, which further necessitates the need for data disaggregation of the API category. Data disaggregation has been identified as a critical health policy issue in addressing health equity within Pasifika, Black, Indigenous, and other communities of color.^53–58^ Although we acknowledge the importance of disaggregating Pasifika from the API umbrella,^36,40,41,43,53^ having a singular monolithic category for Pasifika is equally problematic.^40^ Aggregated data can be harmful to the construction of social welfare and health policies and the development of welfare and health interventions.^40,41,43,59,60^

Tuck and Yang^61^ define data aggregation as a settler move to innocence in which the settler state simultaneously acknowledges health and social disparities experienced by Indigenous communities, but “asterisk” these groups into larger aggregates for the sake of data security or methodological accuracy. Nation states utilize the definition of Indigeneity as a colonial assault to erase Indigenous people, further contributing to their colonial dispossession—allowing settlers to continue to exploit Indigenous lands, resources, and culture.^62^ Thus, aggregation into asterisk groups, or into a singular monolithic category, limits the quality and adequacy of the data and restricts a community's ability to advance data sovereignty efforts for self-determination, decolonization, and to express sovereignty over their bodies, social wellness, and health.^61,63–66^

COVID-19 has brought erasure through data aggregation to the forefront of Pasifika social welfare and public health issues.^39–43^ Parallel historical cases of data erasure during the 1918 influenza pandemic demonstrate how structural racism widened pre-existing health disparities for Black communities—where postpandemic disparities and inaction were attributed to the scarcity of indicator data for social determinants of health.^67^ Furthermore, data aggregation of Pasifika can prevent the tracking of displacement, disease and health disparities, intragroup social inequities (i.e., by ethnicity),^40,41^ and the availability of data on intersectional identities (e.g., gender identities, sexualities, etc.).^68^

### Health equity implications

The diaspora of Pasifika from their native lands to escape poverty driven by displacement and the destruction of Indigenous peoples, lands, and cultures should be of concern to health equity researchers. It is equally important to remember that the assaults on Pasifika continue today and will likely impact future generations of Pasifika in the US.^5,69^ Through settler colonialism, Pasifika are forcibly denied Indigenous futurisms, sovereignty, and self-determination.^5,11,69,70^

Health equity research must take seriously the impact that national and global health emergencies, like the COVID-19 pandemic, has on small populations like Pasifika, especially when COVID-19 incidence and mortality rates are so disproportionate. COVID-19 has impacted Pasifika employment and widened existing health disparities, since employment condition impacts access to food, housing, education, health insurance, and health care resources. Many challenges exist for collecting disaggregated data such as data security related to sample size, the fiscal costs of targeted data collection, and nonstandardized data collection and management practices across state and federal data systems.^40^ However, data should be collected and made available by national data service organizations like the US Census Bureau and state agencies in a way that disaggregates health and social data on Pasifika from data on Asian Americans and better align with data disaggregation policies set out by the Office of Management and Budget.^40,59^

Data should also reflect the sovereignty of Pasifika populations living in the US, such that it disaggregates based on ethnic groups (i.e., Sāmoan, CHamoru, Kānaka Maoli, Marshallese, etc.) and is collected in collaboration with Pasifika community organizations (e.g., Papa Ola Lōkahi, Independent Guåhan, PICA WA). Researchers can support these efforts through community–university partnerships guided by participatory research principles that collect, analyze, and disseminate data for and by the community.^22,71^ Research on smaller Pasifika ethnic communities could also be qualitative in nature to further uncover rich, contextual, and intersectional differences and processes. Engaged community–university partnerships can support the work of community-based organizations to provide health and social services both in national emergencies, such as the COVID-19 pandemic and in the everyday lives of Pasifika communities.

## Abbreviations Used

AI/AN: American Indian and Alaska Native
API: Asian/Pacific Islander
CI: confidence interval
ESD: Employment Security Department
NAOPO: National Association of Pasifika Organizations
NH/PI: Native Hawaiian/Pacific Islander
OFM: Office of Fiscal Management
OR: odds ratio
PICA: Pacific Islander Community Association
PWFH: paid work from home
SES: socioeconomic status
UNAW: ability to work due to COVID
US: United States
WA: Washington state
